# Reducing intradialytic hypotension with intermittent pneumatic compression during haemodialysis: a randomized controlled trial

**DOI:** 10.1093/ckj/sfaf317

**Published:** 2025-10-14

**Authors:** Yixiu Liu, Min Zhi, Min Liu, Ying Huang, Qinjuan Xu, Xiju Luo, Siyan Deng, Jing Chen, Huagang Hu

**Affiliations:** Shanghai First Maternity and Infant Hospital, Pudong New Area, Shanghai, China; School of Nursing, Suzhou Medical College of Soochow University, Suzhou, Jiangsu Province, China; Clinical Medicine Institute of Soochow University and Suzhou BenQ Medical Center, Department of Nephrology, Suzhou BenQ Medical Center, Suzhou, Jiangsu Province, China; Clinical Medicine Institute of Soochow University and Suzhou BenQ Medical Center, Department of Nephrology, Suzhou BenQ Medical Center, Suzhou, Jiangsu Province, China; Hemodialysis Center, First Affiliated Hospital of Soochow University, Suzhou, Jiangsu Province, China; Hemodialysis Center, First Affiliated Hospital of Soochow University, Suzhou, Jiangsu Province, China; School of Nursing, Suzhou Medical College of Soochow University, Suzhou, Jiangsu Province, China; School of Nursing, Suzhou Medical College of Soochow University, Suzhou, Jiangsu Province, China; Hemodialysis Center, First Affiliated Hospital of Soochow University, Suzhou, Jiangsu Province, China; School of Nursing, Suzhou Medical College of Soochow University, Suzhou, Jiangsu Province, China

**Keywords:** intermittent pneumatic compression, intradialytic hypotension, maintenance haemodialysis

## Abstract

**Background:**

Patients undergoing maintenance haemodialysis (MHD) suffer from intradialytic hypotension (IDH), leading to a higher risk of mortality and hospitalization. Intermittent pneumatic compression (IPC) may be a potential intervention to prevent a decrease in blood pressure during haemodialysis (HD). However, evidence about the effects of IPC on intradialytic haemodynamic changes in patients undergoing MHD is lacking. This study aimed to evaluate the effects of IPC on intradialytic haemodynamic changes in patients undergoing MHD.

**Methods:**

In this randomized clinical trial, 30 patients undergoing MHD were randomly assigned to the IPC group (*n* = 14) and the control group (*n* = 16). Participants in the control group received usual care and patients in the IPC group received IPC during all dialysis sessions for 12 weeks. The effects of IPC on haemodynamic indicators were analysed using a generalized estimating equation model.

**Results:**

A total of 26 participants completed the study. The adherence of IPC was 94.2%. The changes in haemodynamic indicators (i.e. blood pressure and heart rate) from pre-dialysis were significantly lower in the IPC group than in the control group at all time points during dialysis (*P* < .05). The incidence of IDH was also lower in the IPC group than in the control group (*P* < .01).

**Conclusion:**

Intradialytic IPC promotes haemodynamic indicators and reduces IDH occurrence in patients undergoing MHD. The compliance and safety of intradialytic IPC were satisfactory. Intradialytic IPC could be implemented in clinical settings to maintain haemodynamic stability during HD.

KEY LEARNING POINTS **What was known:**

- Intradialytic hypotension (IDH) leads to a greater risk of mortality and hospitalization in maintenance haemodialysis (MHD) patients. Intermittent pneumatic compression (IPC) may be a potential intervention to stabilize blood pressure.

**This study adds:**

- Intradialytic IPC promotes haemodynamic stability and reduces IDH occurrence in patients undergoing MHD.

**Potential impact:**

- Intradialytic IPC could be implemented in clinical settings to maintain haemodynamic stability during haemodialysis.

## INTRODUCTION

Intradialytic hypotension (IDH) occurs in ≈8–40% of patients undergoing maintenance haemodialysis (MHD) [1]. IDH could hinder patients from achieving the target ultrafiltration volume [2]. Long-term IDH may cause ischaemia-related complications, including arteriovenous fistula malfunction, decreased residual kidney function and the occurrence of adverse cardiovascular events [2]. Further, IDH is negatively related to quality of life in MHD patients [3].

Current strategies for preventing IDH have some shortcomings. Reducing dialysate temperature may make patients feel chilly [4]. Increasing the sodium concentration in dialysate may cause thirst, weight gain and high blood pressure (BP) [5]. Limiting food intake during dialysis may prevent patients from meeting nutrition needs [6]. It is also difficult for patients to restrict sodium intake [7]. Therefore, it is necessary to find a more effective and patient-friendly way to prevent IDH among MHD patients.

Intermittent pneumatic compression (IPC) can apply intermittent pressure to legs, mimicking the contraction and relaxation of muscles and generating a pressure gradient [8]. Multiple studies have found IPC to be reliable in reducing the incidence of lower limb deep vein thrombosis, reducing the degree of lower limb oedema and alleviating the heaviness and tightness caused by lymphoedema [9–13]. Additionally, IPC was proven to be safe, effective and acceptable among patients [14–18].

Three studies have proven the effectiveness of IPC in IDH prevention, with certain limitations. Álvares *et al.* [19] conducted a study on IPC in patients undergoing MHD. This study has a limited sample size (*n* = 21) and IPC was only used during one dialysis session per participant. Onuigbo [20] reported a case study in which patients were all in poor condition, with excessive body fluids, multiple organ failure and other serious complications. In Miao’s study [21], the lower limb muscle strength of the included patients only achieved a fair level (could perform full-range joint movement but could not resist gravity). One recent study indicated that IPC improved mean arterial pressure (MAP) in 13 elderly people in a seated position, but no other data were available concerning haemodynamic changes during IPC [22, 23]. Therefore, the reliability of the results needs further verification.

Overall, IDH is common and harmful to patients undergoing MHD. Preliminary explorations have been made about the effectiveness of IPC in preventing IDH, but the reliability of the results needs further verification. In addition, relatively little research and insufficient data are available on the effects of IPC on haemodynamics during dialysis. Thus this study aims to investigate the effects of IPC on haemodynamic changes in patients undergoing MHD.

## MATERIALS AND METHODS

### Study design

This study is a randomized controlled trial. Patients undergoing MHD were recruited from the haemodialysis (HD) centre of a tertiary hospital in Suzhou, China, from June to December 2022. This study was conducted in accordance with the Consolidated Standards of Reporting Trials statement [24]. Written consent was obtained from participants prior to participation in the study. Ethical approval was granted by the First Affiliated Hospital of Soochow University (ID: 2021-348). This study is registered in the Chinese Clinical Trial Registry (ChiCTR2200056489).

### Setting and participants

The research was conducted at the First Affiliated Hospital of Soochow University. Patients meeting the eligibility criteria were identified by the research team. The inclusion criteria were age ≥18 years, receiving regular MHD treatment for ≥3 months and voluntarily participating in this study. The exclusion criteria were severe hearing or speech impairment; identified cognitive impairment; systemic diseases such as severe infections, malignant tumours and autoimmune diseases; and contraindication to IPC (e.g. congestive heart failure, deep vein thrombosis, pulmonary embolism, thrombophlebitis and skin abnormalities in the lower limbs).

The sample size of this study was determined by the effect of IPC on systolic blood pressure (SBP), which was the primary outcome of the study. Calculation depended on the G*power 3.1.9.2 package. Before initiating the study we conducted a pilot study involving an IPC group and a control group. Each group including six participants, with each participant undergoing six dialysis sessions over a period of 2 weeks. Based on the data obtained from the pilot study, the effect size (Cohen’s d = 0.20) was obtained by the lowest SBP in the IPC group and the control group. With an effect size of 0.20, power at 80% and independent two-sample t-test at a significant level of 0.05, 394 dialysis sessions for each group were needed. As our study planned to observe 36 dialysis sessions for each patient, 11 patients undergoing MHD were needed. Considering a 20% loss during follow-up, 14 patients were recruited for each group.

### Randomization, allocation concealment and blinding

A simple randomization design was used in this study. An independent research team member was responsible for grouping on an online randomization website (www.sealedenvelope.com). The results were sealed in identical, unmarked and opaque envelopes, then the order of the results was shuffled. Another team member opened the envelopes and the intervention implementer was informed of the assignment of each enrolled patient [25]. Given the active nature of an interventional study, blinding could not be accomplished for participants and assessors.

### Interventions

#### Control group

Participants in the control group received usual care, including routine HD treatment and care. The blood flow rate during dialysis was 200–300 ml/min, the dialysate flow rate was 500 ml/min and bicarbonate dialysate was used in this study. As for the dialysate, the concentration of sodium was 140 mmol/l, the concentration of calcium was 1.5 mmol/l and the concentration of potassium was 2.0–3.0 mmol/l. The area of the polysulfone membrane dialyser was 1.5–1.8 mm^2^. The dry weight for all patients, in both the control and IPC groups, was standardized and managed by a single nephrologist (Y.H.) utilizing the same clinical decision-making framework to ensure homogeneity in fluid management. In this study, dry weight was determined primarily by B-type natriuretic peptide measurements in combination with clinical evaluation (BP trends, interdialytic weight gain and physical examination findings). Bioimpedance and echocardiography were used as supplementary tools when needed. Antihypertensive therapy was prescribed according to a standardized management protocol and participants in both groups maintained the routine use of type and dose of antihypertension drugs. All the patients assumed a supine position during dialysis sessions.

#### IPC group

Based on usual care, IPC was applied throughout each dialysis session. The intervention was completed by the research members. IPC (Kendall SCD Express System, Covidien, Dublin, Ireland) consisted of an SCD RESPONSE controller, a non-disposable tube and a pair of leg cuffs (extending from the ankle to the thigh). IPC pressed the ankle, calf and thigh with a force of 45, 40 and 30 mmHg, respectively, in a sequential, circumferential and gradient manner. IPC followed a cycle of 11 s of compression and 20–60 s of decompression automatically adjusted by the venous refill detection sensor. The compression was given alternatively in each leg. A suitable size of leg cuffs was selected based on the leg circumference and length of each participant. Patients were checked by ultrasonic examination to exclude deep vein thrombosis in the lower limbs before IPC implementation.

### Outcomes

The primary outcome was intradialytic SBP. The second outcomes included intradialytic diastolic blood pressure (DBP), MAP (= 1/3 × SBP + 2/3 × DBP), heart rate, magnitude of BP decreases (maximum decrease in SBP, DBP and MAP), IDH occurrence and safety. IDH is defined in two different ways, namely, IDH_Nadir100_ (the minimum intradialytic SBP <100 mmHg) [26] and IDH_FallSBP20/MAP10_ (a decrease of intradialytic SBP ≥20 mmHg or MAP ≥10 mmHg) [27]. The safety of IPC is defined by IPC-related adverse events such as skin damage and lower limb blood circulation disorders. The acceptance of IPC was assessed using a 10-point Likert scale, ranging from 1 (completely unacceptable) to 10 (completely acceptable), immediately after the final intervention session of the study.

### Data collection

Before dialysis, the BP, heart rate and demographic information of patients (i.e. pre-dialysis weight, dry weight, dialysis modality and medication) were recorded. The BP and heart rate were recorded every 30 minutes during dialysis. The BP was measured using an electronic sphygmomanometer (HBP-1300, Omron, Kyoto, Japan). The heart rate was measured using a pulse oximeter (YX306, Yuyue, Jiangsu, China). In every dialysis session, the BP and heart rate were collected nine times and recorded sequentially as T0 (pre-dialysis) to T8 (240 min after the beginning of dialysis). Dialysis treatment variables, including ultrafiltration volume, ultrafiltration rate, transmembrane pressure, dialysate temperature and blood flow rate, were generated automatically on the dialysis machine. These variables were recorded once per hour. Patients’ intake, IDH-related symptom, treatment and other adverse events were observed and treated. After dialysis, the dry weight of each patient was measured.

Patients’ basic characteristics were recorded on the general information survey and dialysis-related information was recorded on the monitoring table. All the data were provided by patients personally, by the medical staff or collected from the database of the HD centre.

### Statistical analysis

For continuous variables, the Shapiro–Wilk test and quantile–quantile plot were used to examine whether the data presented a normal distribution. Quantitative data following a normal distribution were described by mean and standard deviation (SD). Independent sample t-tests were applied for intergroup comparisons. For quantitative data following an abnormal distribution, median and interquartile range (IQR) were used. Qualitative variables were described in the form of number and percentage. Comparisons between groups were performed using the chi-squared test or Fisher’s exact test. Generalized estimating equations (GEEs) at the individual level with repeated measurements were performed to explore the effects of IPC on the haemodynamics of patients undergoing MHD. All statistical tests were two-tailed and statistical significance was set at 0.05. SPSS 23.0 (IBM, Armonk, NY, USA) was used for analysis.

## RESULTS

### Participant population

**Figure 1:**
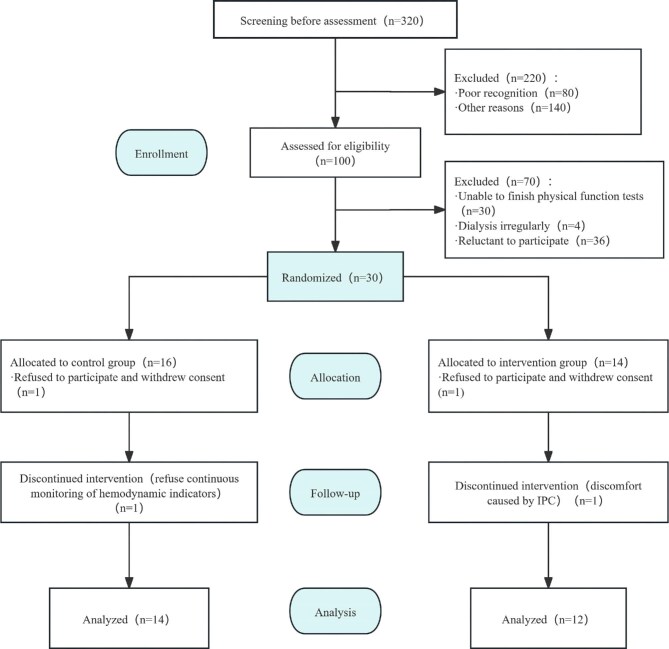
Flow chart of the randomized controlled trial.

Patients were recruited between June and July 2022. A total of 320 patients undergoing MHD were screened, of which 100 were eligible (Fig. 1). A total of 30 patients were enrolled and randomly assigned to the control group (*n* = 16) and the IPC group (*n* = 14). Finally, 26 patients (14 in the control group and 12 in the IPC group) were included in data analysis (Fig. 1).

The baseline characteristics of the participants are presented in Table 1. No statistically significant differences in baseline characteristics were found between the IPC group and the control group (all *P* > .05). Dialysis-related indicators of participants during dialysis are presented in Table 2. The IPC group had higher percentage of interdialytic weight gain/dry weight, ultrafiltration volume, percentage of ultrafiltration volume/dry weight and transmembrane pressure, whereas the control group had higher pre-dialysis DBP, pre-dialysis heart rate, and blood flow (*P* < .05). Given the statistical difference between the two groups, interdialytic weight gain/dry weight served as a confounding factor in the GEE model. No differences were found between groups regarding antihypertensive drug use and food intake.

**Table 1:** Baseline characteristics of participants.

Items	Total (*N* = 26)	IPC group (*n* = 12)	Control group (*n* = 14)	*P*-value
Age (years), mean ± SD	46.9 ± 10.6	46.8 ± 11.8	47.1 ± 9.9	.940
Sex, *n* (%)				1.000^a^
Male	18 (69.2)	8 (44.4)	10 (55.6)	
Female	8 (30.8)	4 (50.0)	4 (50.0)	
Height (cm), mean ± SD	166.7 ± 7.5	165.3 ± 8.5	167.8 ± 6.8	.420
Weight (kg), mean ± SD	61.8 ± 11.9	59.2 ± 9.8	64.0 ± 13.3	.312
Body mass index (kg/m^2^), mean ± SD	22.1 ± 3.2	21.6 ± 2.8	22.6 ± 3.6	.434
Education, *n* (%)				1.000^a^
Junior high school or below	10 (38.5)	5 (50.0)	5 (50.0)	
Above junior high school	16 (61.5)	7 (43.8)	9 (56.3)	
Marriage, *n* (%)				1.000^a^
Unmarried	7 (26.9)	3 (42.9)	4 (57.1)	
Married	19 (73.1)	9 (47.4)	10 (52.6)	
Work status, *n* (%)				.429^a^
Employed	9 (34.6)	3 (33.3)	6 (66.7)	
Unemployed	17 (65.4)	9 (52.9)	8 (47.1)	
Dialysis vintage (months), mean ± SD	89.0 ± 58.9	72.7 ± 53.9	102.9 ± 61.3	.197
Vascular access, *n* (%)				1.000^a^
Arteriovenous fistula	25 (96.2)	12 (48.0)	13 (52.0)	
Central venous catheter	1 (3.8)	0 (0.0)	1 (100.0)	
Monthly personal income (yuan), *n* (%)				.248^a^
≤2000	10 (38.5)	3 (30.0)	7 (70.0)	
>2000	16 (61.5)	9 (56.3)	7 (43.8)	
Smoker, *n* (%)				.330^a^
No	21 (80.8)	11 (52.4)	10 (47.6)	
Yes	5 (19.2)	1 (20.0)	4 (80.0)	
Use erythropoietin, *n* (%)				.365^a^
No	6 (23.1)	4 (66.7)	2 (33.3)	
Yes	20 (76.9)	8 (40.0)	12 (60.0)	
Serum haemoglobin (g/l), mean ± SD	119.3 ± 11.4	118.1 ± 10.7	120.4 ± 12.3	.619
Serum albumin (g/l), mean ± SD	42.0 ± 2.4	42.2 ± 2.2	41.8 ± 2.6	.644
Phosphorus (mmol/l), mean ± SD	2.0 ± 0.6	1.9 ± 0.5	2.1 ± 0.6	.273
Calcium (mmol/l), mean ± SD	2.3 ± 0.2	2.3 ± 0.2	2.3 ± 0.2	.867
Urea (mmol/l), mean ± SD	17.0 ± 3.8	16.8 ± 4.1	17.3 ± 3.6	.727
Uric acid (μmol/l), mean ± SD	368.4 ± 88.8	375.0 ± 88.8	362.6 ± 91.7	.731
Creatinine (μmol/l), mean ± SD	770.7 ± 145.5	764.0 ± 128.3	776.3 ± 163.4	.835
Cause of end-stage renal disease, *n* (%)				.064^a^
Hypertension	6 (23.1)	1 (16.7)	5 (83.3)	
Unknown	7 (26.9)	5 (71.4)	2 (28.6)	
Glomerulonephritis	4 (15.4)	4 (100.0)	0 (0.0)	
Chronic nephritic syndrome	5 (19.2)	3 (60.0)	2 (40.0)	
Other^b^	4 (15.4)	1 (25.0)	3 (75.0)	
Number of comorbidities, mean ± SD	1.0 ± 0.9	0.9 ± 0.5	1.0 ± 1.2	.813
Number of medications, mean ± SD	3.0 ± 1.6	2.5 ± 1.3	3.4 ± 1.8	.156

a Fisher’s exact test.

b Other causes of end-stage renal disease include diabetes, primary glomerular disease, secondary glomerular disease and primary nephrotic syndrome.

**Table 2:** Comparisons of dialysis-related indicators of MHD patients during dialysis sessions.

Indicators	Total (*N* = 936 sessions)	IPC group (*n* = 432 sessions)	Control group (*n* = 504 sessions)	*P*-value
Pre-dialysis SBP (mmHg)	134.65 ± 21.28	133.50 ± 20.91	135.64 ± 21.56	.124
Pre-dialysis DBP (mmHg)	81.58 ± 14.21	80.59 ± 13.52	82.42 ± 14.74	**.047**
Pre-dialysis MAP (mmHg)	99.27 ± 15.69	98.22 ± 15.23	100.16 ± 16.04	.060
Pre-dialysis heart rate (bpm)	80.15 ± 11.67	79.28 ± 9.91	80.89 ± 12.96	**.032**
Antihypertensive medication before dialysis, *n* (%)				.234
No	819 (87.5)	384 (46.9)	435 (53.1)	
Yes	117 (12.5)	48 (41.0)	69 (59.0)	
Food during dialysis, *n* (%)				.332
No	290 (31.0)	127 (43.8)	163 (56.2)	
Yes	646 (69.0)	305 (47.2)	341 (52.8)	
Interdialytic weight gain (kg)	2.27 ± 0.76	2.29 ± 0.73	2.25 ± 0.78	.372
Interdialytic weight gain/dry weight (%)	3.69 ± 1.13	3.92 ± 1.21	3.50 ± 1.02	**<.001**
Ultrafiltration volume (ml)	2382.33 ± 718.03	2442.72 ± 701.08	2330.57 ± 728.96	**.017**
Ultrafiltration volume/dry weight (%)	3.89 ± 1.10	4.16 ± 1.18	3.66 ± 0.97	**<.001**
Ultrafiltration rate (ml/h)	626.02 ± 189.61	627.15 ± 182.45	625.06 ± 195.71	.866
Transmembrane pressure (mmHg)	100.15 ± 75.78	105.52 ± 66.21	95.55 ± 82.90	**.041**
Dialysis fluid temperature (°C)	36.02 ± 1.67	35.93 ± 0.22	36.10 ± 2.26	.113
Blood flow (ml/min)	267.43 ± 18.76	262.77 ± 17.59	271.43 ± 18.82	**<.001**

Data are presented as mean ± SD unless stated otherwise. Statistically significant results are in bold.

### Intervention effects on outcomes

#### Primary outcome: intradialytic SBP

**Figure 2:**
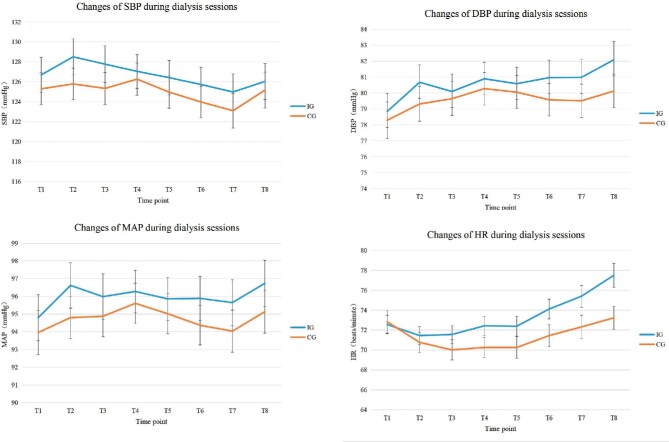
Line chart of haemodynamic indicators during dialysis sessions. T1: 30 minutes after the beginning of dialysis; T2: 60 minutes after the beginning of dialysis; T3: 90 minutes after the beginning of dialysis; T4: 120 minutes after the beginning of dialysis; T5: 150 minutes after the beginning of dialysis; T6: 180 minutes after the beginning of dialysis; T7: 210 minutes after the beginning of dialysis; T8: 240 minutes after the beginning of dialysis; HR: heart rate; IG: intermittent pneumatic compression group; CG: control group.

The changes in intradialytic SBP in the IPC and control groups are shown in Table 3 and Fig. 2. Changes of intradialytic SBP in the IPC group ranged from −5.14 mmHg [95% confidence interval (CI) −6.25 to −4.04] to −8.57 mmHg (95% CI −10.04 to −7.10). Changes in intradialytic SBP in the control group ranged from −9.30 mmHg (95% CI −10.61 to −7.98) to −12.68 mmHg (95% CI −14.08 to −11.28). It can be concluded that the SBP of both groups showed a statistically significant decrease from baseline (*P* < .001). Values of SBP in the IPC group were higher than those in the control group at all time points (*P* < .001 for the group × time interaction effect).

**Table 3:** GEE analysis for haemodynamic effects of IPC in MHD patients.

	Within-group changes from baseline				Differences of between-group changes		
	IPC group		Control group		IPC group vs control group		
	Change (95% CI)	*P*-value	Change (95% CI)	*P*-value	Estimate (95% CI)	*P*-value	*P*-value for time × group interaction effect
SBP							**<.001**
T1	−6.83 (−7.89 to −5.77)	**<.001**	−10.26 (−11.28 to −9.24)	**<.001**	3.40 (1.92–4.87)	**<.001**	
T2	−5.14 (−6.25 to −4.04)	**<.001**	−9.85 (−10.96 to −8.73)	**<.001**	4.67 (3.09–6.24)	**<.001**	
T3	−5.78 (−7.02 to −4.54)	**<.001**	−10.15 (−11.37 to −8.94)	**<.001**	4.34 (2.60–6.07)	**<.001**	
T4	−6.58 (−7.83 to −5.33)	**<.001**	−9.30 (−10.61 to −7.98)	**<.001**	2.68 (0.86–4.49)	**.004**	
T5	−7.12 (−8.36 to −5.88)	**<.001**	−10.59 (−11.96 to −9.23)	**<.001**	3.44 (1.59–5.28)	**<.001**	
T6	−7.83 (−9.21 to −6.44)	**<.001**	−11.59 (−12.97 to −10.21)	**<.001**	3.73 (1.78–5.68)	**<.001**	
T7	−8.57 (−10.04 to −7.10)	**<.001**	−12.68 (−14.08 to −11.28)	**<.001**	4.08 (2.05–6.10)	**<.001**	
T8	−7.62 (−9.14 to −6.10)	**<.001**	−10.64 (−12.12 to −9.17)	**<.001**	2.98 (0.87–5.10)	**.006**	
DBP							**<.001**
T1	−1.75 (−2.51 to −0.99)	**<.001**	−4.09 (−4.75 to −3.44)	**<.001**	2.34 (1.34–3.35)	**<.001**	
T2	0.02 (−0.79–0.82)	.970	−3.12 (−3.85 to −2.39)	**<.001**	3.13 (2.05–4.22)	**<.001**	
T3	−0.52 (−1.38–0.34)	.233	−2.69 (−3.49 to −1.89)	**<.001**	2.17 (0.99–3.34)	**<.001**	
T4	0.25 (−0.60–1.11)	.999	−2.13 (−2.99 to −1.26)	**<.001**	2.38 (1.17–3.60)	**<.001**	
T5	−0.04 (−0.94–0.85)	.924	−2.31 (−3.22 to −1.39)	**<.001**	2.26 (0.98–3.55)	**.001**	
T6	0.32 (−0.61–1.25)	.504	−2.82 (−3.78 to −1.86)	**<.001**	3.14 (1.80–4.47)	**<.001**	
T7	0.31 (−0.67–1.30)	.531	−2.90 (−3.83 to −1.98)	**<.001**	3.22 (1.87–4.57)	**<.001**	
T8	1.44 (0.48–2.40)	**.003**	−2.14 (−3.07 to −1.22)	**<.001**	3.58 (2.25–4.92)	**<.001**	
MAP							**<.001**
T1	−1.76 (−2.34 to −1.17)	**<.001**	−6.12 (−6.81 to −5.43)	**<.001**	2.66 (1.64–3.68)	**<.001**	
T2	−1.71 (−2.51 to −0.90)	**<.001**	−5.37 (−6.14 to −4.59)	**<.001**	3.66 (2.54–4.78)	**<.001**	
T3	−2.28 (−3.15 to −1.41)	**<.001**	−5.17 (−6.02 to −4.31)	**<.001**	2.89 (1.67–4.11)	**<.001**	
T4	−1.94 (−2.83 to −1.05)	**<.001**	−4.52 (−5.47 to −3.58)	**<.001**	2.59 (1.29–3.88)	**<.001**	
T5	−2.41 (−3.33 to −1.49)	**<.001**	−5.12 (−6.11 to −4.13)	**<.001**	2.72 (1.37–4.07)	**<.001**	
T6	−2.40 (−3.37 to −1.42)	**<.001**	−5.75 (−6.77 to −4.72)	**<.001**	3.35 (1.93–4.77)	**<.001**	
T7	−2.63 (−3.66 to −1.59)	**<.001**	−6.24 (−7.27 to −5.21)	**<.001**	3.61 (2.15–5.07)	**<.001**	
T8	−1.59 (−2.63 to −0.54)	**<.001**	−5.15 (−6.19 to −4.10)	**<.001**	3.56 (2.08–5.04)	**<.001**	
HR							**<.001**
T1	−6.75 (−7.29 to −6.20)	**<.001**	−7.98 (−8.56 to −7.41)	**<.001**	1.24 (0.45–2.03)	**.002**	
T2	−7.82 (−8.48 to −7.16)	**<.001**	−10.12 (−10.48 to −9.39)	**<.001**	2.31 (1.33–3.29)	**<.001**	
T3	−7.68 (−8.42 to −6.94)	**<.001**	−10.82 (−11.66 to −9.98)	**<.001**	3.15 (2.03–4.27)	**<.001**	
T4	−6.79 (−7.55 to −6.03)	**<.001**	−10.67 (−11.58 to −9.77)	**<.001**	3.89 (2.71–5.07)	**<.001**	
T5	−6.87 (−7.67 to −6.07)	**<.001**	−10.64 (−11.59 to −9.68)	**<.001**	3.78 (2.53–5.02)	**<.001**	
T6	−5.11 (−5.97 to −4.25)	**<.001**	−9.45 (−10.38 to −8.53)	**<.001**	4.35 (3.08–5.61)	**<.001**	
T7	−3.82 (−4.79 to −2.85)	**<.001**	−8.46 (−9.38 to −7.54)	**<.001**	4.65 (3.32–5.98)	**<.001**	
T8	−1.62 (−2.69 to −0.54)	**<.001**	−7.59 (−8.52 to −6.66)	**<.001**	5.98 (4.56–7.40)	**<.001**	

T1: 30 minutes after the beginning of dialysis; T2: 60 minutes after the beginning of dialysis; T3: 90 minutes after the beginning of dialysis; T4: 120 minutes after the beginning of dialysis; T5: 150 minutes after the beginning of dialysis; T6: 180 minutes after the beginning of dialysis; T7: 210 minutes after the beginning of dialysis; T8: 240 minutes after the beginning of dialysis.

Statistically significant results are in bold.

#### Secondary outcomes: intradialytic DBP, MAP, heart rate, IDH frequency, BP decrease, IPC complian**ce, safety and acceptability**

The changes in intradialytic DBP, MAP and heart rate in the IPC and control groups are presented in Table 3 and Fig. 2. Changes of intradialytic DBP in the IPC group ranged from −1.75 mmHg (95% CI −2.51 to −0.99) to 1.44 mmHg (95% CI 0.48–2.40). Changes in intradialytic DBP in the control group ranged from −4.09 mmHg (95% CI −4.75 to −3.44) to −2.13 mmHg (95% CI −2.99 to −1.26). Changes in intradialytic MAP in the IPC group ranged from −1.59 mmHg (95% CI −2.63 to −0.54) to −2.63 mmHg (95% CI −3.66 to −1.59). Changes in intradialytic MAP in the control group ranged from −6.24 mmHg (95% CI −7.27 to −5.21) to −4.52 mmHg (95% CI −5.47 to −3.58). Changes in intradialytic heart rate in the IPC group ranged from −7.82 bpm (95% CI −8.48 to −7.16) to −1.62 bpm (95% CI −2.69 to −0.54). Changes in intradialytic heart rate in the control group ranged from −10.82 bpm (95% CI −11.66 to −9.98) to −7.59 bpm (95% CI −8.52 to −6.66). The DBP, MAP and heart rate in both groups decreased from baseline (*P* < .001). Values of DBP and MAP in the IPC group were greater than those in the control group (*P* < .001 for the group × time interaction effect). The value of heart rate in the control group was greater than that in the IPC group at T1 without statistical significance, while from T2 to T8, the values in the IPC group were greater than those in the control group (*P* < .001 for the group × time interaction effect).

Overall, IDH occurred less frequently in the IPC group than in the control group (*P* < .01; Supplementary Table S1). The incidence rates of IDH_Nadir100_ during dialysis were 12.7% and 21.0% in the IPC and control groups, respectively (*P* = .001). In addition, the incidence rates of IDH_FallSBP20/MAP10_ during dialysis were 50.2% and 65.7% in the IPC and control groups, respectively (*P* < .001). The maximum decreases in SBP, DBP and MAP were lower in the IPC group than in the control group (*P* < .001; Supplementary Table S1).

The overall completion rate of IPC was 94.2% of sessions. In 4.2% of sessions, patients used the device for <3.5 h, which was the specified time to achieve better results. The detailed performances and reasons for poor compliance are provided in Supplementary Table S2. No skin damage or ischaemia occurred during IPC applications. In addition, fewer IDH-related symptoms and other adverse events occurred in the IPC group than its counterpart, demonstrating the safety of IPC as well (*P* < .01; Supplementary Table S3). Participants’ acceptability ratings for the IPC on a 10-point scale were as follows: 7 (*n* = 3), 8 (*n* = 2), 9 (*n* = 2) and 10 (*n* = 5).

## DISCUSSION

The results indicate that the haemodynamic stability of the IPC group was better than that of the control group. Both groups experienced a decrease at the beginning of dialysis, demonstrating a great haemodynamic change at the initiation of dialysis. SBP may be more sensitive to dialysis and more difficult to recover from. In addition, the results of this study indicated that IDH occurred less frequently and the maximum decrease in BP was lower in the IPC group than in the control group.

Few topics related to the haemodynamic effects of IPC have been previously discussed. In a previous study, during seated rest, compression resulted in a 4.5 ± 6.5 mmHg increase in MAP among older adults. Data were collected from 13 adults (4 males) >65 years of age [22]. The result corresponds with our study of MAP. However, the study involved a posture transition, complicating the change in BP. In another study enrolling 50 healthy volunteers ages 23–35 years who were free of systemic disease, BP was measured 15 s prior to, at onset and after IPC inflation [23]. The IPC device has been proven to have no significant impact on non-invasive BP measurement (i.e. SBP, DBP and MAP) of the ankle [23]. Subjects and time points of BP measurement may explain the difference between outcomes.

IPC may improve haemodynamic stability by sequentially pressing the lower limbs, forming a pressure gradient, increasing venous blood flow and velocity and accelerating blood circulation [28, 29]. In a study, the venous volume of IPC with calf-and-foot-included leg cuffs was greater than that of IPC with foot-included leg cuffs (3–3.5 times) and calf-included leg cuffs (2–2.5 times). The same study also indicated that increasing pressure during IPC compression rarely improved blood flow [9]. Another study showed that IPC significantly increased the velocity and volume flow whenever the limbs are elevated, horizontal or dependent. Moreover, positions made a difference in the measurement [11]. A randomized crossover trial conducted by Tai et al. [30] showed IPC to be ineffective in intradialytic haemodynamic parameters including central blood volume. The potential factors influencing the haemodynamic effects of IPC require further exploration.

During IPC, no skin damage occurred. Also, fewer IDH-related symptoms and other adverse events were observed. Considering compliance and safety, the intervention is feasible. In other studies that applied IPC in patients undergoing MHD, adverse effects were rarely reported [19, 20, 31].

The study has some strengths. First, several points ensured the safety of the intervention. The study excluded patients with congestive heart failure or other conditions that could easily increase cardiac output and burden. In addition, patients were given an ultrasound examination to exclude lower extremity deep vein thrombosis. During the intervention, patients were instructed to wear comfortable, breathable, lightweight pants to reduce friction on the skin. Patients only used IPC during dialysis under the supervision of medical staff and researchers and their skin and blood flow conditions were monitored and evaluated. Also, patients were frequently asked about their feelings. Second, some actions were taken to ensure the effective application of IPC. During the research, IPC was used throughout the dialysis process, with a high frequency of haemodynamic indicator measurement (once every 30 minutes), which increased the probability of detecting haemodynamic instability. Patients’ legs were placed flat on the dialysis bed, which reduced bending or folding of the tube.

However, several limitations should be considered when interpreting the findings. First, the limited sample size may weaken the generalizability of this study. However, our study included 36 dialysis sessions per participant, which satisfied the predetermined sample size requirement based on power calculations. Second, the nature of the intervention made it impossible to blind participants, researchers and assessors, which may have introduced expectation bias. To mitigate this, this study employed standardized data collection protocols and validated measurement instruments to ensure methodological consistency. Third, the IPC device and its usage process need to be improved. Due to varying individual acceptance levels, equipment costs and a shortage of medical personnel, it may be difficult to make full clinical use of the equipment. Nevertheless, this study yields novel evidence supporting its practical applicability in real-world settings.

In conclusion, IPC may be effective in promoting intradialytic haemodynamic stability and in reducing IDH occurrence and BP decreases among patients undergoing MHD. During intervention, IPC was found to be safe and tolerable. However, further research is necessary to investigate the long-term efficacy of IPC and its direct impact on critical outcomes in MHD patients, including physical function, laboratory parameters and health-related quality of life.

## Supplementary Material

sfaf317_Supplemental_File

## Data Availability

The data that support the findings of this study are not publicly available due to their containing information that could compromise the privacy of research participants, but are available from the corresponding author upon reasonable request.

## References

[1] Kanbay M, Ertuglu LA, Afsar B et al. An update review of intradialytic hypotension: concept, risk factors, clinical implications and management. Clin Kidney J 2020;13:981–93. 10.1093/ckj/sfaa078 33391741 PMC7769545

[2] Intradialytic Hypotension Prevention and Treatment Expert Working Group, Renal and Blood Purification Committee, Chinese Medicine Education Society . [Expert consensus on prevention and treatment of intradialytic hypotension in hemodialysis (2022)]. Zhonghua Nei Ke Za Zhi 2022;61:269–81. 35263968 10.3760/cma.j.cn112138-20210601-00384

[3] Zhi M, Zeng Y, Chen C et al. The relationship between intradialytic hypotension and health-related quality of life in patients undergoing hemodialysis: a cross-sectional study. Sci Rep 2025;15:11532. 10.1038/s41598-025-96286-y 40185881 PMC11971372

[4] Mustafa RA, Bdair F, Aki EA et al. Effect of lowering the dialysate temperature in chronic hemodialysis: a systematic review and meta-analysis. Clin J Am Soc Nephrol 2016;11:442–57. 10.2215/CJN.04580415 26712807 PMC4791810

[5] Flythe JE, Causland FRM. Dialysate sodium: rationale for evolution over time. Semin Dial 2017;30:99–111. 10.1111/sdi.12570 28066913 PMC5334180

[6] Kalantar-Zadeh K, Ikizler TA. Let them eat during dialysis: an overlooked opportunity to improve outcomes in maintenance hemodialysis patients. J Ren Nutr 2013;23:157–63. 10.1053/j.jrn.2012.11.001 23313434 PMC3632653

[7] Ok E . How to successfully achieve salt restriction in dialysis patients? What are the outcomes? Blood Purif 2010;29:102–4. 10.1159/000245633. 20093812

[8] Torres C, Fuentes HE, Saadaldin H et al. Intermittent pneumatic compression in patients with ESRD. A systematic review. Hemodial Int 2019;23:433–44. 10.1111/hdi.12771 31283096

[9] Zhang D, Li F, Li X et al. Effect of intermittent pneumatic compression on preventing deep vein thrombosis among stroke patients: a systematic review and meta-analysis. Worldviews Ev Based Nurs 2018;15:189–96. 10.1111/wvn.12288 29729658

[10] Tessari M, Tisato V, Rimondi E et al. Effects of intermittent pneumatic compression treatment on clinical outcomes and biochemical markers in patients at low mobility with lower limb edema. J Vasc Surg Venous Lymphat Disord 2018;6:500–10. 10.1016/j.jvsv.2018.01.019 29909855

[11] Tastaban E, Soyder A, Aydin E et al. Role of intermittent pneumatic compression in the treatment of breast cancer–related lymphoedema: a randomized controlled trial. Clin Rehabil 2020;34:220–8. 10.1177/0269215519888792 31795748

[12] Pavon JM, Adam SS, Razouki ZA et al. Effectiveness of intermittent pneumatic compression devices for venous thromboembolism prophylaxis in high-risk surgical patients: a systematic review. J Arthroplasty 2016;31:524–32. 10.1016/j.arth.2015.09.043 26525487

[13] Nelson EA, Hillman A, Thomas K. Intermittent pneumatic compression for treating venous leg ulcers. Cochrane Database Syst Rev 2014;2014:CD001899. 24820100 10.1002/14651858.CD001899.pub4 PMC10788769

[14] Ren W, Duan Y, Jan Y-K et al. Effect of intermittent pneumatic compression with different inflation pressures on the distal microvascular responses of the foot in people with type 2 diabetes mellitus. Int Wound J 2022;19:968–77. 10.1111/iwj.13693 34528370 PMC9284627

[15] Delis KT, Slimani G, Hafez HM et al. Enhancing venous outflow in the lower limb with intermittent pneumatic compression. A comparative haemodynamic analysis on the effect of foot vs. calf vs. foot and calf compression. Eur J Vasc Endovasc Surg 2000;19:250–60. 10.1053/ejvs.1999.1048 10753688

[16] Lurie F, Awaya DJ, Kistner RL et al. Hemodynamic effect of intermittent pneumatic compression and the position of the body. J Vasc Surg 2003;37:137–42. 10.1067/mva.2002.24 12514591

[17] Labropoulos N, Leon LR Jr, Bhatti A et al. Hemodynamic effects of intermittent pneumatic compression in patients with critical limb ischemia. J Vasc Surg 2005;42:710–6. 10.1016/j.jvs.2005.05.051 16242559

[18] Kakkos SK, Szendro G, Griffin M et al. Improved hemodynamic effectiveness and associated clinical correlations of a new intermittent pneumatic compression system in patients with chronic venous insufficiency. J Vasc Surg 2001;34:915–22. 10.1067/mva.2001.118822 11700495

[19] Álvares VRC, Ramos CD, Pereira BJ et al. Pneumatic compression, but not exercise, can avoid intradialytic hypotension: a randomized trial. Am J Nephrol 2017;45:409–16. 10.1159/000471513 28407637

[20] Onuigbo MA . Bilateral lower extremity sequential compression devices (SCDs): a novel approach to the management of intra-dialytic hypotension in the outpatient setting—report of a case series. Ren Fail 2010;32:32–5. 10.3109/08860220903367478 20113263

[21] Miao L . Effects of passive movement on maintenance hemodialysis patients with intradialytic hypotension. Beijing: Peking Union Medical College, 2013.

[22] Zuj KA, Hedge ET, Milligan JD et al. Intermittent compression of the calf muscle as a countermeasure to protect blood pressure and brain blood flow in upright posture in older adults. Eur J Appl Physiol 2021;121:839–48. 10.1007/s00421-020-04547-7 33386985

[23] Parikh BR, Simon AM, Kouvaras JN et al. Influence of intermittent pneumatic compression devices on non-invasive blood pressure measurement of the ankle. J Clin Monit Comput 2007;21:381–6. 10.1007/s10877-007-9100-1 17972148

[24] Schulz KF, Altman DG, Moher D. CONSORT 2010 statement: updated guidelines for reporting parallel group randomised trials. J Pharmacol Pharmacother 2010;1:100–7. 10.4103/0976-500X.72352 21350618 PMC3043330

[25] Doig GS, Simpson F. Randomization and allocation concealment: a practical guide for researchers. J Crit Care 2005;20:187–91. 10.1016/j.jcrc.2005.04.005 16139163

[26] Kuipers J, Oosterhuis JK, Krijnen WP et al. Prevalence of intradialytic hypotension, clinical symptoms and nursing interventions—a three-months, prospective study of 3818 haemodialysis sessions. BMC Nephrol 2016;17:21. 10.1186/s12882-016-0231-9 26922795 PMC4769826

[27] Flythe JE, Xue H, Lynch KE et al. Association of mortality risk with various definitions of intradialytic hypotension. J Am Soc Nephrol 2015;26:724–34. 10.1681/ASN.2014020222 25270068 PMC4341481

[28] Chen AH, Frangos SG, Kilaru S et al. Intermittent pneumatic compression devices—physiological mechanisms of action. Eur J Vasc Endovasc Surg 2001;21:383–92. 10.1053/ejvs.2001.1348 11352511

[29] Sparrow RA, Hardy JG, Fentem PH. Effect of ‘antiembolism’ compression hosiery on leg blood volume. Br J Surg 1995;82:53–9. 10.1002/bjs.1800820120 7881957

[30] Tai DJ, Ahmed SB, Palacios-Derflingher L et al. Pneumatic compression devices during hemodialysis: a randomized crossover trial. Nephrol Dial Transplant 2013;28:982–90. 10.1093/ndt/gfs502 23136215

[31] Dang MH, Sia C, Fernando S et al. Pneumatic compression devices in prevention of intradialytic hypotension. Int J Nephrol Kidney Fail 2019;5:1–5.

