# Supplementary material for: Reducing intradialytic hypotension with intermittent pneumatic compression during haemodialysis: a randomized controlled trial
Source: Clin Kidney J. 2025 Oct 14;18(11):sfaf317. doi: 10.1093/ckj/sfaf317 (PMC12612675; doi:10.1093/ckj/sfaf317)
Supplement: sfaf317_Supplemental_File [file sfaf317_supplemental_file.docx]

Table S1 Comparisons for intradialytic hypotension occurrence and blood pressure decline of maintenance hemodialysis patients (*n* = 936 sessions)

| Indicators | Control group  (*n* = 504 sessions) | IPC group  (*n* = 432 sessions) | *P*-value |
| --- | --- | --- | --- |
| IDH_Nadir100_, *n* (%) |  |  | **0.001** |
| No | 398 (79.0) | 377 (87.3) |  |
| Yes | 106 (21.0) | 55 (12.7) |  |
| IDH_FallSBP20/MAP10_, *n* (%) |  |  | **<0.001** |
| No | 173 (34.3) | 215 (49.8) |  |
| Yes | 331 (65.7) | 217 (50.2) |  |
| Maximum decrease in SBP, mmHg, mean ± SD | 21.6 ± 13.9 | 17.9 ± 12.9 | **<0.001** |
| Maximum decrease in DBP, mmHg, mean ± SD | 10.0 ± 9.7 | 7.4 ± 8.9 | **<0.001** |
| Maximum decrease in MAP, mmHg, mean ± SD | 13.0 ± 10.4 | 9.8 ± 9.3 | **<0.001** |

Abbreviations: IPC, intermittent pneumatic compression; SBP, systolic blood pressure; DBP, diastolic blood pressure; MAP, mean arterial pressure; SD, standard deviation; IDH_Nadir100,_ the minimum of intradialytic SBP < 100mmHg; IDH_FallSBP20/MAP10_, a decrease of intradialytic SBP ≥ 20mmHg or a decrease of intradialytic MAP ≥ 10 mmHg; Numbers in bold represent statistically significant results.

Table S2 Compliance of maintenance hemodialysis patients applied with intermittent pneumatic compression (*n* = 432 sessions)

| Poor compliance performance and reasons | *n* (%) |
| --- | --- |
| Refusal of using device |  |
| Synovitis | 19 (4.4) |
| Involuntary leg twitching | 6 (1.4) |
| Using device for less than 3.5 hours |  |
| Device breakdown | 1 (0.2) |
| Early dialysis termination | 2 (0.5) |
| Discomfort caused by application | 1 (0.2) |
| Later use of the machine | 14 (3.2) |

Abbreviations: IPC, intermittent pneumatic compression; MHD, maintenance hemodialysis.

Table S3 The effect of intermittent pneumatic compression on intradialytic hypotension related symptoms and interventions or other adverse effects during dialysis

| Indicators | IPC group  (*n* = 432 sessions) | Control group  （*n* = 504 sessions） | *χ^2^* | *P*-value |
| --- | --- | --- | --- | --- |
| Intradialytic hypotension related symptoms |  |  | 0.419 | 0.517 |
| No | 388（46.5） | 446（53.5） |  |  |
| Yes | 44（43.1） | 58（56.9） |  |  |
| Intradialytic hypotension related interventions |  |  | 10.944 | **<0.001** |
| No | 403（48.0） | 437（52.0） |  |  |
| Yes | 29（30.2） | 67（69.8） |  |  |
| Other adverse effects |  |  | 8.012 | **0.005** |
| No | 419（47.2） | 468（52.8） |  |  |
| Yes | 13（26.5） | 36（73.5） |  |  |
